# New products

**DOI:** 10.1155/S1463924692000373

**Published:** 1992


Journal of Automatic Chemistry, Vol. 14, No. 5 (September–October 1992), pp. 193–197

### New products

### Computer-controlled pump

Index Instruments Ltd, of Ramsey, Huntingdon, have announced their entry into an entirely new market with the launch of the Indexpump 300. The new pump is designed for sampling, dosing or metering applications and operates as a stand-alone unit or under computer control.

Using a virtually pulse-free peristaltic pump head, the Indexpump 300 can accommodate single or dual tubes. An alternative version is also available for use with auto-analysers having up to 20 channels. Being simple to program and easy to use, this new pump is very versatile. It is especially useful for cell filling applications, where complete purging is important. It can be programmed to operate twice in every cycle with a wait time between operations. This has been shown to give better purging than a single operation.

The pump head takes any standard tubing from 1·5 mm to 8 mm bore, and has a variable drive over the range of 10 rpm to 100 rpm. There are also both time and volume dispensing modes. Accuracy is from 0·01 ml to 1 ml depending on tube size. A built-in RS232 communication port provides the means for connection to a computer, allowing the pump to be set and operated remotely if required.

*Further information from Index Instruments Ltd, Bury Road Industrial Estate, Ramsey, Huntingdon, Cambridgeshire PE17 1NA, UK. Tel.: 0487 814313; fax: 0487 812789.*

### Saccharimeter

Unrefined, dark sugar samples have previously required treatment with lead acetate solution (which can present environmental risks) to clarify them before analysis. Optical Activity has developed a dual wavelength, high performance Saccharimeter which can measure dark sugar samples accurately and without the need for lead acetate pretreatment.

The instrument operates at either the standard wavelength of 589·44 nm or in the near infra-red region at approximately 880 nm. Sugar solutions do not absorb infra-red radiation and so the concentration of even the darkest samples can be determined quickly and precisely.

The SacchAAr 880 is the first instrument of its kind to incorporate a dual wavelength measuring system. It combines accurate analysis with minimal sample preparation. Filtration, to remove suspended solids, is the only pretreatment required. When samples measured at 589·44 nm have a transmission of less than 3% of the source beam, an LCD automatically warns the user to switch to the infra-red wavelength – this is achieved by turning a knob. Measurements are shown on the instrument's liquid crystal display in either °Z, or temperature-compensated °Z to an accuracy of ±0·01. In addition to the LCD display, the SacchAAr 880 also has outputs for connecting external printers, computers or other data collection equipment.

A major benefit of the SacchAAr 880 is the ease with which the calibration can be checked and, if necessary, adjusted on-site using an existing quartz plate and a single, clear sugar solution, of *any* concentration. However, the SacchAAr 880 does not normally require any routine recalibration.

The SacchAAr 880 accepts all internationally standard sample cells, including micro and flow-through cells. It also has a built-in multiplier facility to compensate for measurements made with non-standard cells, or different concentration solutions; in addition, it can be connected to a refractometer to provide direct readout of apparent sugar purity.

*Further information on the SacchAAr 880 is available from Optical Activity Ltd, Bury Road Industrial Estate, Ramsey, Huntingdon PE17 1NA, UK. Tel.: 0487 813913, fax: 0487 812789.*

### HP 1090 HPLC system

Hewlett-Packard has announced the HP 1090 Win, the company's latest addition to its HP 1090 family of high performance liquid chromatography instruments. Designed for analysts with sophisticated separation or method development requirements, especially where their work is subject to Good Laboratory Practice (GLP) guidelines, the system features Microsoft Windows (Microsoft Corporation) application software for 3-D data handling.

The HP 1090 Win liquid chromatography system allows multiple analytes to be accurately quantified and qualified in the same injection. It incorporates a diode-array detector, data from which can be used to confirm quantification results using retention time and spectral information.

HP believes it is unique in offering an HPLC system that can control and acquire data from UV/visible absorbance and fluorescence LC detectors, while providing a full range of software tools for GLP method verification. Among the available techniques are peak qualification, based on absorbance ratios of two or more simultaneously acquired signal wavelengths; peak purity calculations based on comparisons of UV/visible absorbance spectra recorded during elution; and positive peak identification using electronically archived UV/visible absorbance or fluorescence spectra from spectral libraries.

All separation, detection, data evaluation and quantification parameters can be specified using a single method. The actual instrument performance is tracked by the system during the run, and details stored with the raw data in a 'register file'. This contains all the information needed to reconstruct a particular analysis, as may be required during GLP regulatory audits in the pharmaceutical and food industries and environmental monitoring labs.

The controlling HPLC3D ChemStation is based on an IBM-compatible personal computer running under MS-DOS (Microsoft Corporation) with the Microsoft Windows 3.1 graphical user interface. It can be used with third-party word-processing, spreadsheet, database management and electronic mail software; and may be connected to local area networks using HP ChemLan products.

The HP 1090 Win system's flow path is designed for optimum separation performance using narrow-bore and high speed columns. Narrow-bore separations improve detection in trace level analyses, while high speed columns improve throughput for large numbers of similar samples. Sample preparation can be automated by the system's autosampler, for example to improve a method's selectivity by chemical derivatization.

The system comprises a ternary gradient pump module, autosampler module and diode-array detection module together with an HP Vectra 486s/20 PC, HP LaserJet IIIP printer and HPLC3D ChemStation operating software.

*Readers' enquiries to Verena Haller, Hewlett-Packard SA, 150 Route du Nant-d'Avril, CH 1217 Meyrin (GE) 2, Switzerland. Tel.: 44 81 567 8670.*

### QC expert software

Controlling the quality of the results generated during an ICP-MS or ICP-AES analysis is both labour-intensive and important. If unacceptable data is reported, samples may have to be reanalysed. Perkin-Elmer has developed QC Expert, a quality-control software program, which controls both the instrument and the autosampler, automating the quality control protocols required to monitor ICP analyses. During the analysis, if the quality of the data produced is unacceptable according to user-defined limits, pre-established actions are taken to restore quality.

Analytical parameters are divided into two kinds of file. The Analytical Method File contains information on calibration standards, calibration algorithms, QC standards and allowable limits. The Sample Description File contains information specific to the individual sample. These combine to produce an Autosampler Worksheet file containing the analytical sequence, which can be edited by the user. Formal reports can be generated, data exported to third-party software programs and long-term data quality and trends can also be monitored.

*For further information contact Perkin-Elmer Ltd, Post Office Lane, Beaconsfield, Buckinghamshire HP9 1QA, UK. Tel.: 0494 676161.*

### Atomic absorption system

The fully automated 5100 ZL Atomic Absorption system is optimized for flame, furnace and mercury/hydride sampling. It combines conventional flame and furnace AA with Zeeman-corrected graphite furnace analysis. A transversely-heated graphite tube design with integrated electrical contacts and an integrated L'vov-type platform uses the longitudinal Zeeman background correction system with magnetic field parallel to the optical light beam to maintain analytical accuracy.

For increased productivity and convenience, up to 12 elements can be determined automatically using a single method with optimum conditions for each.

The system is controlled by an industry-standard PC using specialized AA software with advanced graphics display capabilities, complete data storage, built-in quality control and report generation procedures.

*For further information contact Perkin-Elmer Ltd, Beaconsfield, Buckinghamshire HP9 1QA, UK. Tel.: 0494 676161.*

### Laboratory glassware washing

Suitable for use in laboratory facilities of all sizes, Dawson MMP Ltd's

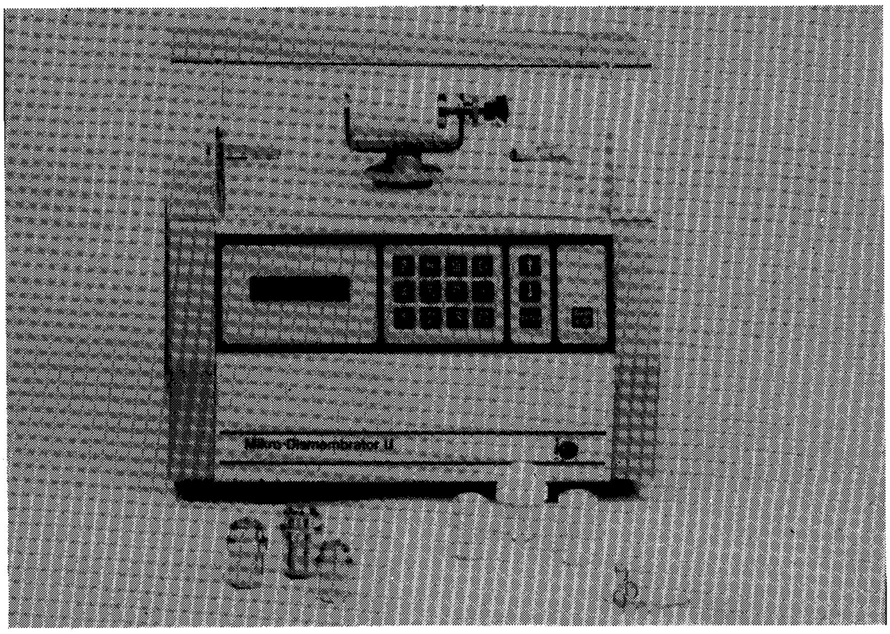

*B. Braun Biotech's upgrade of the Micro-Dismembrator II—the Micro-Dismembrator U. The new laboratory-scale vibration grinding mill provides several valuable features including an integral countdown timer, variable shaking frequency, a digital keypad and display and a transparent isolating cover for maximum safety. The mill can homogenize, disperse, dissolve, extract, emulsify, grind, pulverize and mix organic as well as inorganic sample material. Applications include the grinding of teeth, bones and inorganic materials or organisms in toxicology, disruption of plants for trace element analysis in environmental research, disintegration of soft tissue for histology and disruption of micro-organisms, yeast cells or tissue cell cultures in biotechnology. The unit can also be used to shake microtitre plates.*

*Details from B. Braun Biotech, 13–14 Farmborough Close, Aylesbury Vale Industrial Park, Aylesbury, Buckinghamshire HP20 1DG, UK. Tel.: 0297 393900.*

glassware washing machines are designed to safeguard standards of cleanliness and as an aid to improved working efficiency. The range includes the LG100 front-loading machine, which has a five-stage programmable wash cycle comprising pre-wash, main wash, neutralizing rinse, rinse and demineralized final rinse. The programme cycle, cycle time and temperature can be adjusted to suit specific applications. Continuous cycle monitoring employs a series of indicator lamps to keep the operator informed of programme status. Also capable of disinfection by advancing the temperature control, the LG100 is fitted with a large purpose-built integral water softener offering total regeneration, so any risk of bacterial build-up is eliminated by full backwash and purge functions on every regeneration.

An optional dryer cabinet, designated TD60 is available and is designed to compliment the LG100.

The MD200D washer/disinfector offers the convenience of washing and drying facilities in a single machine with obvious space-saving advantages. It is designed to provide high standards of performance in the washing and disinfection of all kinds of laboratory glassware, and has the additional capability of being able to process a wide range of medical apparatus. Like the LG100, the MD200D also features a five-stage programmable wash cycle and in-cycle monitoring system. The time and temperature controls are infinately variable and the drying facility can be overridden if required.

The use of robust stainless-steel construction throughout the range ensures the appliances are able to withstand the rigours of constant use with minimal routine maintenance. Versatility of both the LG100 and MD200D machine is enhanced by the extensive range of baskets and inserts available. Manufactured from 18/8 chrome nickel steel the baskets are designed to hold a wide variety of equipment.

*Details from John Crispin, Dawson MMP Ltd, Wath Road, Elsecar, Barnsley, South Yorkshire S74 8HJ, UK. Tel.: 0226 350450; fax: 0226 340384.*

### Guide to sampling VOC air pollution

A Thermal Desorption Applications Note, reviewing sampling procedures for volatile organic compounds (VOCs) in ambient air, is available without charge from Perkin-Elmer. It summarizes the applications and limitations of various sample collection methods, including passivated canisters, pumped sampling onto sorbent tubes, on-line air stream sampling and diffusive monitoring.

*For further information, contact Perkin-Elmer Ltd (as above).*

### NIR integrating sphere accessory

Perkin-Elmer's Near-IR Integrating Sphere is a solid sampling accessory for the 2000 FT-IR. It provides a convenient method for Near-infrared Diffuse Reflectance (NIDR) measurements of a range of powders and granular materials. The integrating sphere provides a large sample area, which reduces sampling error in measurements of inhomogeneous materials. NIDR is of particular interest in the food and pharmaceuticals industries for quality assurance and analytical applications, as it permits measurement of most samples undiluted and in their original state. Some common applications include wheat grains, flours, milk powder, pharmaceutical tablets, powdered chemicals and geological samples.

*For further information contact Perkin-Elmer Ltd (as above).*

### Perkin-Elmer system 2000

A range of liquid sampling accessories from Axiom is now available for the Perkin-Elmer Sytem 2000 FT-IR.

FT-IR Tunnel cells are designed for quantitative liquid sampling, and are the first cylindrical internal reflectance sampling accessories to provide the high degree of absorbance linearity and repeatability required for quantitative analysis. Several versions are available, suitable for laboratory and process monitoring applications.

The Dipper 210 permits analysis of a wide variety of samples with virtually no sample handling. Consisting of a conical ATR sensing head and articulated sample region mount, the probe can be dipped into any open container.

*For further information contact Perkin-Elmer Ltd (as above).*

### Accessories for the Lambda 19

The Perkin-Elmer Lambda 19 UV/VIS/NIR is a high-performance double-beam, double-monochromator spectrometer controlled by an industry-standard PC. A range of six new reflectometry accessories, manufactured by Labsphere, Inc. increase the performance and versatility of the Lambda 19 for measurement of most solid samples:

The Drata 19 150 mm integrating sphere has a wavelength of 200–2500 nm and can measure samples in both diffuse transmittance and reflectance.

The Large Sample Compartment accessory allows measurement in transmittance of samples up to 32 × 32 × 10 cm and any size sample in the reflectance mode.

The Long Path Transmittance acccessory accommodates optical component samples up to 32 cm long and 15 cm in diameter.

The Remote Fibre Optic Sphere accessory uses a 100 mm integrating sphere with fibre optic cable to bring the spectrometer to the sample.

The 0°/45° Reflectance accessory is ideal for specular light-excluded colour measurements.

The Variable Angle Absolute Specular Reflectance/Transmittance accessory allows variable angle transmission measurement of translucent samples.

*For further information contact Perkin-Elmer Ltd (as above).*

### Laboratory data management

Adept Scientific has announced GRAMS/386, brand-new Windows-based data management and pro-

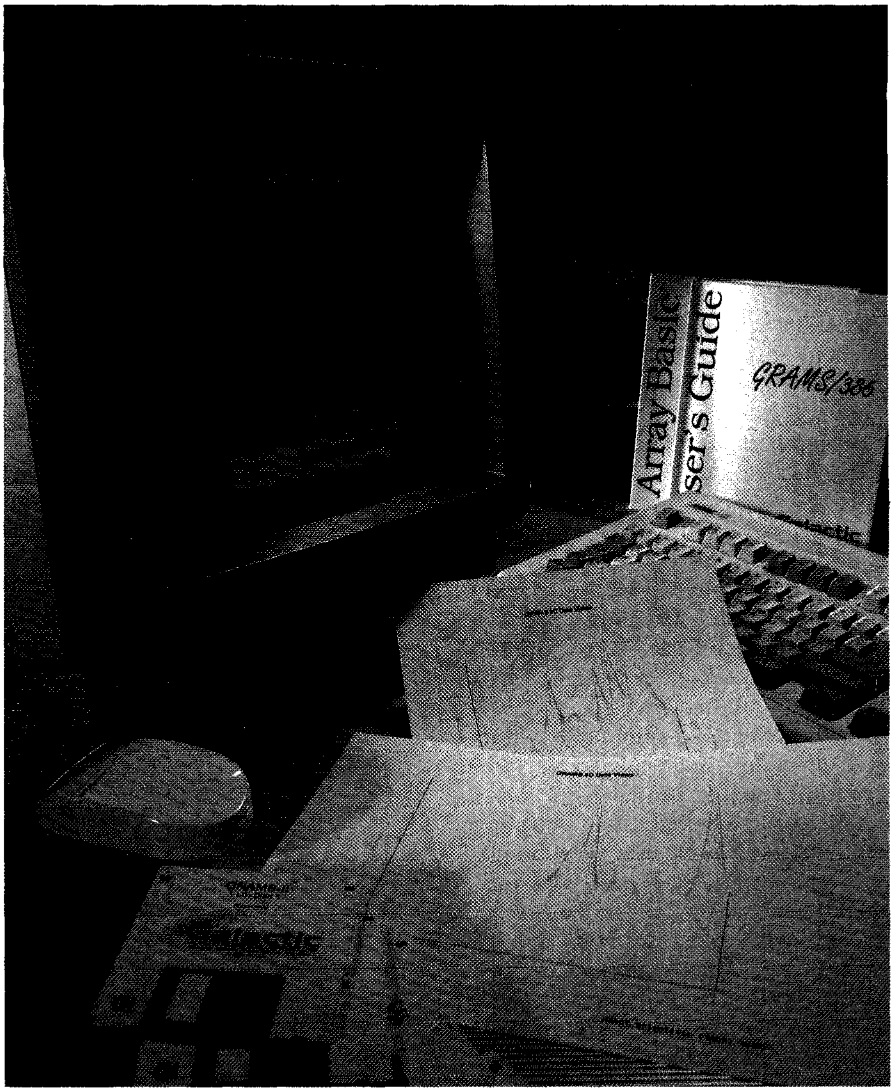

*Manipulation of three-dimensional data in GRAMS/386.*

cessing software from Galactic Industries. Designed to cope with the large amounts of data produced in today's laboratory, GRAMS/386 is available in three levels of functionality to meet the needs of every laboratory. Compatible with modern analytical instruments from a variety of manufacturers, GRAMS/386 offers users the opportunity to view and process all their data, whatever the source, within a single package. This eliminates the need to purchase separate, and usually very different, data handling packages from individual instrument manufacturers.

Like Galactic's established Spectra Calc and Lab Calc software (still available and fully supported by Adept Scientific for non-Windows users), GRAMS/386 can acquire, import, manipulate and organize data from a variety of analytical instruments. GRAMS – Graphical Relational Array Management System – takes full advantage of the Windows user interface to streamline data viewing and processing chores within a single, powerful software environment. With GRAMS/386, data handling is faster, more efficient and independent of instrument manufacturers' own highly specific data processing software.

With GRAMS/386, the entire laboratory data output is instantly accessible. All experimental results can be saved or retrieved with a click of the mouse. Data files created with Spectra Calc and Lab Calc are fully compatible with GRAMS/386. Presentation-quality graphics and WYSIWYG plotting allow the user to incorporate plots and charts into reports.

Rather than constraining the user to system-specific software, the GRAMS database transparently organizes gigabytes of spectroscopic, chromatographic and other types of data from most modern analytical instruments. A comprehensive range of fast, accurate data processing routines includes curve-fitting, subtraction, quantitative analysis, peak picking, integration and numerous chemometric procedures.

Array Basic, Galactic's powerful programming language, allows users to modify existing GRAMS/386 data handling algorithms, or create new ones to meet the requirement of specific experiments.

There are three options: GRAMS Level I supports file and library import/export, a data viewer, the Array Basic language and library and cut-and-paste between Windows applications as well as a basic database; GRAMS Level II adds a dynamic data exchange (DDE) interface to Windows for data acquisition; and GRAMS Level III, the network database version, incorporates a powerful, three-dimensional, fully relational database to provide complete laboratory data management in a single package.

GRAMS/386 requires a 386 or 486 personal computer with maths coprocessor, minimum 2 MByte memory, VGA or better display, hard disk, mouse and Windows 3·0 or higher.

*Further information from Neil Chapman, Adept Scientific Micro Systems Ltd, 6 Business Centre West, Avenue One, Letchworth, Hertfordshire SG6 2HB, UK. Tel.: 0462 48005.*

### IR correlation spectra

Galactic Industries' 2DIR runs under Lab Calc. The program takes advantage of recent advances in commercial step-scan FT-IR instrumentation to simplify the analysis of time-resolved spectroscopic responses of a sample to sinusoidal perturbations. Experiment responses of this type are traditionally analysed using phase-sensitive-detection devices such as two phase lock-in amplifiers. Two examples of such modulation experiments are dynamic infra-red linear dichroism (DIRLD) and photoacoustic spectroscopy (PAS).

When a quadrature, or two-phase lock-in amplifier, is tuned to the perturbation frequency being applied to the sample, two signals are measured which contain all of the information relevant to the time-resolved spectral response of the sample. These signals are typically collected into two data files: one which is in-phase with the sample modulation, and a second which is 90° out of phase, or in quadrature, with the modulation. The information contained in these in-phase and quadrature spectra can be represented in a two-dimensional map after a correlation analysis is performed. The 2D representation of the data emphasizes the correlation in response of one wavenumber in the spectrum relative to any other wavenumber. Representing the data in a 2D map can enhance the spectral resolution if different spectral components of a broad band contour respond to the applied perturbation out of phase with each other.

The 2DIR program takes advantage of the array operations of Galactic's powerful language interpreter, Array Basic, to calculate the correlations row by row rather than point by point, allowing the application to run considerably faster. The program is designed so that correlation calculations can be performed for experiments run at different times or within different regions of the spectrum. 2DIR will work with any type of data containing in-phase and quadrature phase spectra. In some cases, data from two different types of spectroscopy can be cross correlated. Two dimensional maps are then generated with one data type along one axis and another data type along the other.

2DIR runs under Lab Calc software on PCs, ATs, PS/2s, and compatibles.

*For more information contact Kelly W. McIntire, Galactic Industries Corporation, 395 Main Street, Salem, New Hampshire 03079, USA. Tel.: 603 898 7600.*

### Printing weighing data

The AD-8121 multi-function printer from A&D Instruments provides all necessary information to meet Good Laboratory Practice requirements. The printer supplies a permanent printed record in many laboratory applications, such as formulation weighing, monitoring changes in weight during chemical storage, checkweighing and statistical analysis.

The AD-8121 is an impact dot matrix printer which will accept RS-232C and current loop input from any A&D electronic balance, counting balance, platform scale or weighing indicator. It is compact and lightweight for easy portability, and can be AC or battery-operated for use anywhere.

Users of the AD-8121 have access to a full range of statistical functions: weight and total weight data, counting and total counting, number of operations, standard deviation, all variations on date and time. The interval printing option takes weight readings at any of seven pre-selected intervals. Chart printing can then be used to display graphically changes in weight, plus the interval, start and stop times.

*For further information contact A&D Instruments Ltd, Abingdon Science Park, Abingdon, Oxon OX14 3YS, UK. Tel.: 0235 550420.*

